# The Adrenal Gland of Squamata (Reptilia): A Comparative Overview

**DOI:** 10.3390/ani13172686

**Published:** 2023-08-22

**Authors:** Anna Capaldo

**Affiliations:** Department of Biology, University of Naples Federico II, Via Cinthia, Edificio 7, 80126 Naples, Italy; anna.capaldo@unina.it or nevotta16@libero.it

**Keywords:** adrenal gland, chromaffin tissue, NE/E cell ratio, Reptilia, Squamata, steroidogenic tissue

## Abstract

**Simple Summary:**

The adrenal gland plays a fundamental role in the physiology of vertebrates, regulating hydrosaline balance, carbohydrate and lipid metabolism, and stress response. This enables living vertebrates to deal with both internal and environmental stress stimuli. The adrenal gland consists of steroidogenic tissue, which synthesizes steroid hormones, and chromaffin tissue, which mainly produces catecholamines, norepinephrine, and epinephrine. The degree of separation between the two types of tissue, the compactness of the adrenal gland, and its topographical relationships with the kidney have changed over the course of evolutionary history. In most reptiles, the glands have close relationships with the gonads and genital ducts. In the main order of Reptilia called Squamata (e.g., lizards and snakes), the gland shows extreme variability in the organization between chromaffin and steroidogenic tissues and in the numerical ratio between norepinephrine and epinephrine cells. This variability reflects the relationship between the two adrenal tissues, which, in turn, could be related to the phylogenetic history of the species. This overview examines the general characteristics of the adrenal glands of the squamates and the different structural patterns in the different species belonging to this order.

**Abstract:**

The adrenal gland is a complex endocrine organ composed of two components: a steroidogenic tissue, which produces steroid hormones, and a chromaffin tissue, which mainly produces norepinephrine and epinephrine. Through evolution, their relationships with each other changed. They begin as isolated chromaffin and steroidogenic cell aggregates, typical of fish, and end with the advanced compact gland, typical of mammals, which consists of an external steroidogenic cortical zone and an internal chromaffin medullary zone. The adrenal gland of reptiles is unique because, with few exceptions, it is near the gonads and genital ducts, and the chromaffin and steroidogenic tissues are closely associated. However, the degree of mixing is variable. For example, in Squamata, the mixing degree of chromaffin and steroidogenic tissues, their reciprocal position in the gland, and the relative quantities of norepinephrine and epinephrine secreted by the chromaffin cells are extremely variable. This variability could be related to the phylogenetic history of the species. After a brief discussion of the adrenal gland and its main functions in vertebrates, this overview will examine the general characteristics of the adrenal gland of squamates, the differences in morphology of the gland, and the possible relationships with the phylogeny of the different species.

## 1. The Adrenal Gland

**Figure 1:**
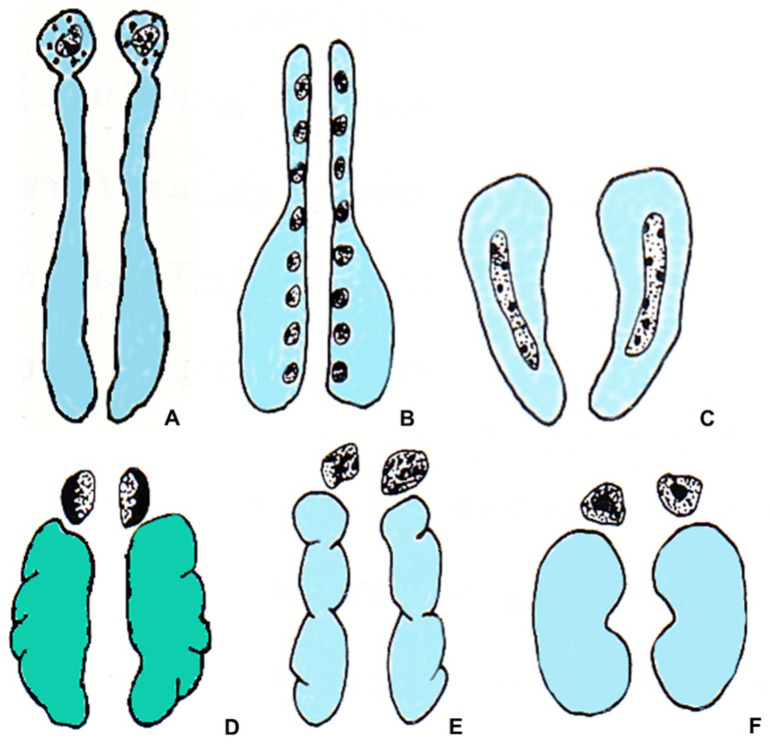
Schematic drawing showing the relationships between the steroidogenic tissue (gray), the chromaffin tissue (black), the kidneys (light blue), and the gonads (green) in vertebrates. (**A**) teleost fish; (**B**) urodele amphibian; (**C**) anuran amphibian; (**D**) lacertilian reptile; (**E**) bird; (**F**) mammal.

All vertebrates have chromaffin tissue, derived from neural crests, which mainly produces the catecholamines norepinephrine (NE) and epinephrine (E), and steroidogenic tissue, of mesodermal origin, which produces steroid hormones. However, the topographical relationships between these two tissues and their own anatomical positions are different in different groups of vertebrates. During evolution, a trend toward developing a close anatomical relationship between these two types of tissue can be observed. The two tissues are separated in hagfish and lampreys, and only occasionally are the chromaffin cells located near the steroidogenic cells. The chromaffin cells can be found within the heart and in the great veins returning blood to the heart [1], and clusters of steroidogenic cells are associated with the posterior cardinal veins and the mesonephric parenchyma [2,3]. Also, in cartilaginous fishes, the steroidogenic and the chromaffin tissues are separated, and their organization is different in different species. In some elasmobranchs and holocephalans, there is one unpaired adrenal gland, made up of adrenocortical cells and placed between the posterior ends of the kidneys. However, in other species, the steroidogenic tissue forms paired strands along the medial border of the posterior kidney and small islets on the anterior surface of the kidneys [3]. The chromaffin cells are associated with the paravertebral autonomic ganglia, called auxiliary bodies [1]. In bony fish, the distribution and localization of the steroidogenic and chromaffin tissues and their relationships with the kidney differ in the various species. In teleosts, depending on the species, steroidogenic cells are present in the head kidney and are frequently associated with the dorsal posterior cardinal veins [3]. The chromaffin cells can be found in the walls of the posterior cardinal veins and the head kidney, where they may be single or may form groups of several cells, separate from steroidogenic cells, or intermingled with them [1,3] (Figure 1).

In amphibians, the organization of the steroidogenic and chromaffin tissues in urodeles differs from those of anurans. In urodeles, the steroidogenic tissue forms many islets, also containing sporadic chromaffin cells. The islets are scattered on the ventral surface of the mesonephric kidney, close to its medial margin, and are separated from each other. The degree of association between the chromaffin and the steroidogenic cells is variable. In Salamandridae and Plethodontidae, the two types of cells are mixed [1,4]. In anurans, the steroidogenic tissue located on the ventral surface of the kidneys forms continuous strands containing clusters of chromaffin cells. In both urodeles and anurans, the degree of compactness of the gland and the aggregation of steroidogenic and chromaffin cells is variable. Generally, it increases in the transition from basal to advanced families [1,5]. Moreover, in ranid anurans, in addition to the steroidogenic and chromaffin cells, a third type of cell has been found, the summer or Stilling cells, only present in summer and with a still unknown function [3].

In reptiles, the steroidogenic and the chromaffin tissues form a discrete gland that is in contact with the gonads and genital ducts, except for chelonians, where the gland is in contact with the ventral surface of the kidney [6]. Although steroidogenic and chromaffin tissues are generally associated, there are considerable variations between species in their distribution. It is not possible to present a valid general picture for the whole class [2,7]. A discrete gland located near the kidneys is also observed in birds and mammals. In birds, the adrenal gland may be two separate structures in contact with each other or a single median one. The steroidogenic tissue forms radially arranged cords mixed with blood vessels and chromaffin cell groups [8]. Finally, in mammals, the steroidogenic tissue forms the peripheral portion of the gland, called the cortex, surrounding an inner part formed by the chromaffin tissue, called the medulla, an arrangement allowing a considerable degree of mixing at the border between the two tissues (Figure 1) [1,2,3,7].

According to Grassi Milano [6], the different types of organization of the adrenal gland in amniotes (reptiles, birds, and mammals) are congruent with the main phyletic lines of this group. In chelonians, having a “diffused” structural pattern, the adrenal gland is placed on the ventral surface of the kidney, as in anuran amphibians. Such a condition, in which the steroidogenic cells and the chromaffin cells are mixed together, is considered plesiomorphic in comparison to other amniotes. In the other amniotes, the adrenal gland has a “compact” structural pattern, considered apomorphic. Moreover, in amniotes, three conditions can be observed in the relationships existing between the steroidogenic and the chromaffin cells: (1) The typical condition of Rhynchocephalia and Squamata, in which the chromaffin cells are present on the dorsal region of the gland; (2) the typical condition of Crocodilia and birds, in which the chromaffin cells are dispersed among the steroidogenic cells; and (3) the typical condition of mammals (except prototherians), in which the chromaffin cells form an inner medulla, surrounded by a steroidogenic cortex.

The different anatomical positions of the gland, which is made up of steroidogenic and chromaffin tissues, justify the different nomenclature of this gland in different vertebrate groups. Indeed, when the gland is located between the kidneys, as in amphibians, it is normally referred to as interrenal. Additionally, when the gland, as in most reptiles, does not have an anatomical relationship with the kidney but with gonads and genital ducts, it is preferred to call it the adrenal gland. In mammals, the gland is called suprarenal because it is generally found on the cephalic pole of the kidney. However, the term adrenal is so widespread that it is generally used, regardless of position [2].

The adrenal gland plays an important role in controlling glucose and lipid metabolism, regulating the saline water balance, and responding to stress, enabling living organisms to react to emergency situations. Indeed, the steroidogenic tissue mainly produces glucocorticoid and mineralocorticoid hormones. Glucocorticoids, such as corticosterone and cortisol, are produced under the control of the hypothalamic corticotropin-releasing hormone (CRH) and the pituitary adrenocorticotropic hormone (ACTH). These steroid hormones are involved in glucose metabolism, affect lipid accumulation and mobilization, have anti-inflammatory action, are necessary for the growth and differentiation of different organs, and are involved in the stress response. Mineralocorticoid hormones, such as aldosterone, are generally produced under the control of the renin–angiotensin system or potassium levels and are mainly involved in the control of saline water balance. However, in different groups of vertebrates, the main hormones produced and their relative quantities may be slightly different, so their activities may overlap in some cases [3]. The chromaffin tissue produces NE and E (the same molecules released as neurotransmitters by the sympathetic system) under the control of the sympatho–adreno–medullary system. However, different species differ in the main type of catecholamine produced. Moreover, the chromaffin cells can synthesize and store many neuropeptides, neurotransmitters, and cytokines that can be co-released with catecholamines. Catecholamines regulate lipid and glucose metabolism, blood pressure, and thermoregulation in homeothermic animals and play a key role in responding to stressful situations [9,10,11,12,13].

## 2. Paracrine Relationships in the Adrenal Gland

**Figure 2:**
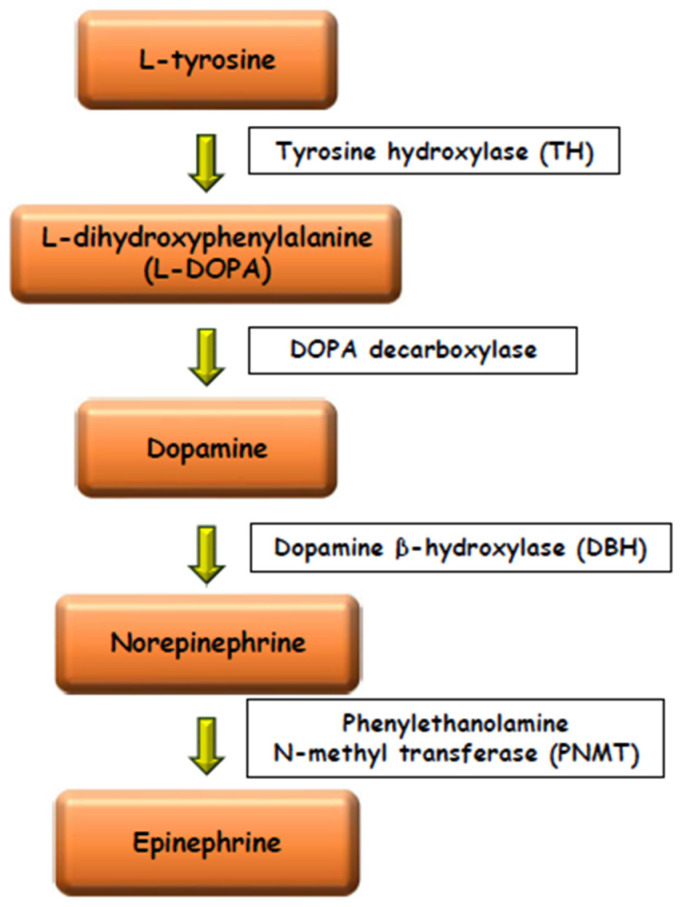
Schematic drawing of the catecholamines biosynthetic pathway.

Numerous experimental data have shown the existence of paracrine relationships between the steroidogenic and the chromaffin tissues. In mammals glucocorticoids stimulate the activity of the tyrosine hydroxylase (TH) enzyme, the rate-limiting enzyme in catecholamine biosynthesis [14,15] and the phenylethanolamine-N-methyltransferase (PNMT) enzyme, the last of the catecholamines biosynthetic pathway, converting NE into E [16,17,18,19,20] (Figure 2). Aldosterone elevates PNMT activity too, but with less power than glucocorticoids [21].

Glucocorticoids are believed to upregulate catecholamine synthesis, storage, and secretion [20]. Moreover, they are essential for postnatal maintenance of adrenal and extra-adrenal chromaffin cells [22]. In birds, the adrenocorticotropic hormone (ACTH) influences TH enzyme [15], increases PNMT activity and the conversion of NE into E via intraglandular glucocorticoid production [19,23]. In reptiles, as in mammals, a stimulating action of glucocorticoids on the enzyme PNMT has been found [24,25,26] whereas in fish and amphibians such stimulating action has not been observed [1,27,28]. In turn, catecholamines and the neuropeptides co-released with them from the chromaffin tissue, may influence the synthesis and release of the steroid hormones from the steroidogenic tissue in a paracrine way. This paracrine relationship has been observed in many vertebrates, including fish and amphibians [1,14]. The resulting crosstalk that settles between the two components of the gland may be important to synchronize the response of this gland to stress [29]. From this point of view, the evolutionary tendency to the progressive coalescence of steroidogenic and chromaffin tissues has optimized the ability of the organisms to respond to stressful situations and supports the widely accepted opinion that the progressive concentration of the glandular tissues has been a selectively favorable process [30].

## 3. The Morphology of the Chromaffin and the Steroidogenic Tissues

The morphology of the chromaffin and the steroidogenic tissues can be studied with techniques utilized in light and electron microscopy. As for chromaffin tissue, it should be noted that two situations have been observed: the presence of two types of chromaffin cells, one of which contains NE and the other E, or the presence of only one type of chromaffin cell that produces both catecholamines. The first type of organization is generally present: (a) in fishes; (b) in anurans amphibians, where NE and E cells are usually intermingled; (c) in reptiles, where the distribution of NE and E cells is different in the different species; and (d) in birds, where the chromaffin cells are usually mixed and do not have any preferential location. As regards urodele amphibians, two subtypes of chromaffin cells were observed [4], whereas in *Triturus carnifex*, a single type of chromaffin cell was found, producing both NE and E, correlated to the environmental temperature [31,32] and the reproductive cycle [33]. In mammals, generally NE and E cells have been found, but their relative quantity and their topographical location are different in different species. For example, about 75% of the chromaffin cells in humans and bovine animals, and 80–85% in the rat, are E cells. In the rat, but not in bovine animals, the NE cells are generally located at the boundary between the cortex and the medulla, whereas in other species the E cells are peripherally located [2,11,34,35,36].

**Figure 3:**
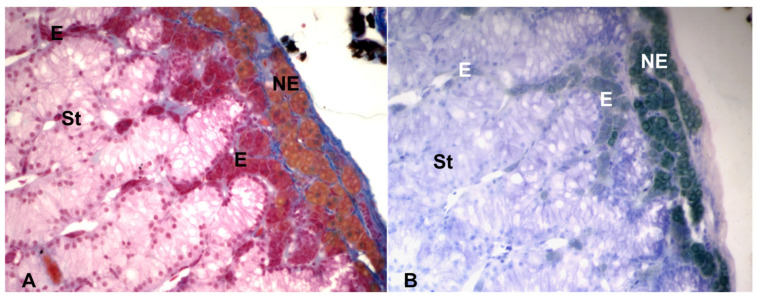
Light microscopy of the adrenal gland of the lacertid, *Podarcis siculus*. (**A**) Mallory stain, showing the NE cells gold yellow, the E cells red and the steroidogenic (St) tissue light pink. (**B**) Giemsa stain, showing the NE cells dark green and the E cells light green. Magnification: 400×.

The two types of chromaffin cells can be distinguished with histochemical staining, using a fixative based on potassium dichromate, the Wood’s fixative, a mixture of 2.5% potassium dichromate and 1% sodium sulphate (buffered at pH 4.1 with 5 M acetate buffer) and 10% formaldehyde. Indeed, the oxidation with potassium dichromate produces brown insoluble pigments from both NE and E, which can be subsequently distinguished using two histochemical stains: the Wood stain and the Giemsa stain [37,38]. The Wood stain, a mixture of eosin and aniline blue, buffered at pH 4 with 5 M acetate buffer, stains the NE cells gold and the E cells orange red. A similar staining is also obtained with a trichromic histological stain, the Mallory staining, with which the NE cells appear gold yellow, and the E cells appear red. Moreover, this staining has in addition the advantage of staining well also the steroidogenic tissue, that appears light pink. Finally, the Giemsa solution, modified according to Pearse [38], a mixture of eosin blue and methylene blue, stains the NE cells dark green and the E cells light green [25,26] (Figure 3).

**Figure 4:**
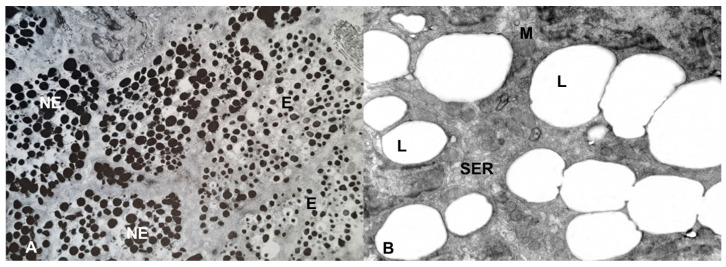
Electron microscopy of the adrenal gland of the lacertid, *Podarcis siculus*. (**A**) the NE cells have electron-dense, polymorphic granules, whereas the E cells have roundish, moderately electron-dense granules, characterized by a clear halo between the core and the limiting membrane. Magnification: 5000×. (**B**) the cytoplasm of the steroidogenic cells is mainly characterized by lipid (L) droplets, smooth endoplasmic reticulum (SER) and mitochondria (M). Magnification: 4000×.

When only one type of chromaffin cell, synthesizing both catecholamines, is present, the histochemical stains produce a mixed staining. In that case, the best method to study the chromaffin cells is electron microscopy, with which the simultaneous presence of the two types of granules in the same cell, as well as the presence of separate NE and E chromaffin cells, can be observed. Indeed, such a distinction can be obtained by using a first fixation with glutaraldehyde followed by a post-fixation with osmium tetroxide. Glutaraldehyde induces the formation of an insoluble complex with NE, which will be subsequently stained with osmium tetroxide. On the contrary, E is largely lost during the fixation and dehydration because it does not form such a complex. Therefore, E granules show only a light electron-density [39]. Under the electron microscope, the NE granules appear polymorphic and very electron-dense, with a core closely adhering to the limiting membrane, whereas the E granules are roundish, moderately electron-dense and characterized by a clear halo between the core and the limiting membrane [40,41] (Figure 4). A study, performed in E-deficient mice, generated by knocking out PNMT gene, has recently shown that the E granules still retain their shape and general appearance, despite the lack of E [42].

However, the chromaffin cells can also be studied with many other histological, histochemical and immunocytochemical methods [43,44]; for example, the NE cells can be identified through the immunopositivity to the enzyme, tyrosine hydroxylase (TH), converting l-tyrosine to l-3,4-dihydroxyphenylalanine (l-DOPA) (Figure 2), and by staining with Harris hematoxylin after treatment with citrate buffer at pH 6 [36]. The E cells possess the enzyme PNMT, converting NE into E, and therefore can be identified through the immunopositivity to this enzyme. An exception is represented by birds, in which all chromaffin cells possess the enzyme PNMT, but it is active only in the E cells. Therefore, in birds the PNMT enzyme cannot be used to discriminate the E cells from the NE cells [19].

Under light microscopy, the steroidogenic tissue is characterized by cells with a generally weakly stained and finely spongy cytoplasm, due to the presence of lipid droplets, composed of esters of cholesterol, the precursor of steroid hormones (Figure 3). At the electron microscopic level, the steroidogenic cells are generally characterized by a poor rough endoplasmic reticulum and few free ribosomes while they are rich in smooth endoplasmic reticulum, Golgi complex and mitochondria, having inner membranes forming tubular or vesicular cristae. Moreover, the steroidogenic cells have lipid droplets, the quantity of which varies according to the species and the functional stage of the tissue [45] (Figure 4). In non-mammalian vertebrates, the steroidogenic tissue is usually arranged in cords forming interconnected networks, without preferential organization. However, in birds and in some reptiles, just below the connective capsule, the cords of steroidogenic cells assume a ring or basket arrangement at the periphery of the gland, and parallel more deeply, suggesting a functional zonation, that has been observed in birds [8,46,47] and reptiles, in which the existence of several sub-populations of adrenocortical cells is supposed [8,48]. In mammals, the adrenal cortex is organized in an outer glomerular zone, mainly producing mineralocorticoid hormones; a deep fasciculata zone, mainly producing glucocorticoid hormones, and an inner reticularis zone, mainly producing androgen hormones. However, this standard model of adrenocortical zonation may be variable; in the rat, for example, a white, or undifferentiated zone (ZU) was observed between glomerular and fasciculata zones. The ZU zone was considered a stem/progenitor cell zone [49], but its presence in other species is not clear [50].

## 4. The NE/E Cell Ratio

The possibility of distinguishing NE cells from E cells by light microscopy makes it possible to calculate the numerical ratio between NE and E cells, or NE/E cell ratio. This ratio reflects the proximity relationships between the steroidogenic and the chromaffin tissues, since, in mammals, birds and reptiles, glucocorticoids stimulate the activity of the enzyme PNMT, that methylates NE converting it into E (Figure 2). Therefore, high E levels indicate an efficient methylation process due to a close contact between the chromaffin and the steroidogenic cells, and therefore high glucocorticoid levels available to stimulate the enzyme PNMT [29]. The values of this ratio are very variable in birds and reptiles, and attempts were made to attribute a phylogenetic significance to its variations. For example, it was found that birds with more primitive ancestry, as cormorant and egret, had more NE, while recently evolved species, as passerine birds, had more E; a NE/E cell ratio around 1/1 was typical of species, as pigeon and cuckoo, having an intermediate evolutionary position [51]. However, this opinion is no more supported by recent bird phylogenetic reconstructions [52]. Also in reptiles, the NE/E cell ratio is highly variable. For example, in Squamata (lizards, snakes and amphisbaenians), high values of this ratio correspond to a high degree of separation between the steroidogenic and the chromaffin tissues, whereas low values of this ratio correspond to a higher degree of admixing of the two tissues. As for birds, a correlation between the value of NE/E cell ratio and the phylogenetic history of different species was assumed [2,51]. This correlation was confirmed by numerous biochemical, karyological and paleontological data [53,54,55,56]. However, in recent years the hypotheses on Squamata phylogeny have changed radically, especially if both morphological and molecular data are considered [57,58,59,60,61,62]. Therefore, the hypothesis of a correspondence between the value of the NE/E cell ratio and the greater or lesser antiquity of the species in Squamata should be verified with further studies.

## 5. The Adrenal Gland of Reptiles

Before describing the adrenal gland of reptiles, it must be emphasized that, from a systematic point of view, there is no agreement on the taxon Reptilia, since morphological and molecular studies have questioned its composition and its nomenclature [63]. The discussion on the systematic position and the nomenclature of this taxon goes beyond the scope of this paper, in which the traditional and better-known term of “reptiles” will still be used. Moreover, it must be said that in this review are considered also papers published many years ago, relating to species whose nomenclature has changed over time. Where possible, the current nomenclature in brackets right after the specific name, utilized in the mentioned papers, will be quoted [64].

In reptiles, the steroidogenic and the chromaffin tissues are associated to form a gland in close relationship with the gonads and the genital ducts (Figure 1), except for chelonians, where the gland is related to the ventral surface of the kidney. The degree of admixing of the two tissues is highly variable and it is not possible to define a scheme valid for all reptiles [1,2,7]. Indeed, in Chelonia and Crocodilia, a close intermingling of the steroidogenic and the chromaffin cells, without any concentration of chromaffin tissue on the dorsal region of adrenal gland, can be observed [6,65,66]. The arrangement of the adrenal gland of Chelonia, recently considered diapsid reptiles (for review see [63]), is like that of amphibians, forming a stripe on the ventral surface of the kidney, whereas the arrangement of the adrenal gland of Crocodilia, characterized by strands of chromaffin cells intermingled with interrenal cords, is like that of birds, where the chromaffin tissue forms clumps or strands of cells mixed with blood vessels and interrenal steroidogenic cords, both in subcapsular zone and inner part of the gland [6]. In Rhynchocephalia and Squamata, the chromaffin tissue is mainly distributed on dorsal part of the gland. The adrenal gland of Rhynchocephalia has a parenchyma of steroidogenic tissue also containing islets of chromaffin cells; a major part of chromaffin tissue forms a dorsal mass that sends expansions into the steroidogenic parenchyma, whereas some clusters of chromaffin cells are also present on the ventral surface of the gland [2,6,67].

## 6. The Adrenal Gland of Squamata

In Squamata (lizards, snakes and amphisbaenians), the general arrangement of the adrenal gland is like that of Rhynchocephalia; indeed, the adrenal gland shows a steroidogenic parenchyma and a dorsal mass made of chromaffin tissue. The latter is often present also in the parenchyma, where it forms islets, more or less numerous, having few chromaffin cells. Unlike Rhynchocephalia, usually the adrenal gland of Squamata does not show chromaffin tissue on the ventral surface of the gland [65,66,67,68,69]. However, there are many differences in the distribution of the chromaffin cells, which can form a continuous envelope around the steroidogenic parenchyma, with few or no inner islets, as well as they can be concentrated to form islets scattered in the parenchyma and a very reduced dorsal mass. Above all, this great variability in the distribution of the two types of tissue can be observed not only between different families, but also within the same family or within the same genus, as has been observed studying many species belonging to different families, subfamilies, orders, and infraorders (for review see [2]). These morphological variations are accompanied by corresponding variations in the NE/E cell ratio; the morphology of adrenal gland of some of the species studied is illustrated below.

### 6.1. Lacertidae

**Figure 5:**
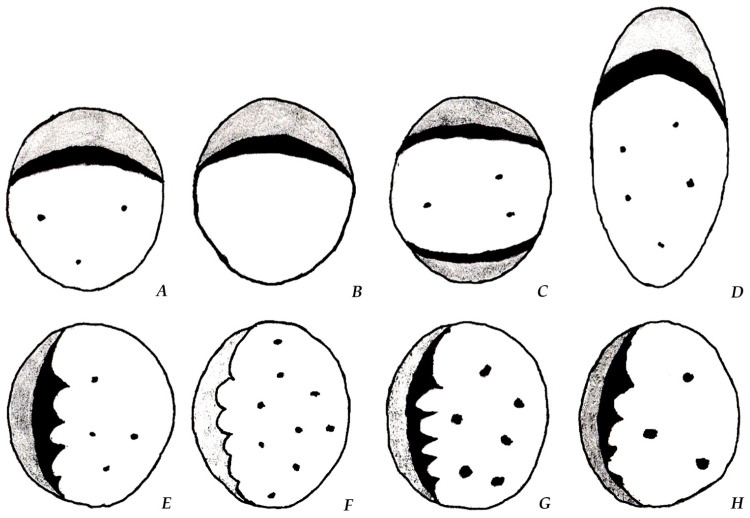
Schematic drawing showing the different organization of NE cells (grey), E cells (black) and the steroidogenic tissue (white) in the adrenal gland of (**A**) *G. galloti*; (**B**) *L. graeca* (*H. graeca*); (**C**) *L. dugesii* (*T. dugesii*); (**D**) *L. pater* (*T. pater*); (**E**) *L. lepida* (*T. lepidus*); (**F**) *L. trilineata*; (**G**) *L. schreiberi*; (**H**) *L. viridis*.

The family Lacertidae includes more than 250 species with wide geographical distribution [70]. Studies performed on different species belonging to this family have found an extensive variation in the structure of adrenal gland and the distribution of steroidogenic and chromaffin tissues, also in the same genus [2,71,72]. An example can be provided by the study of species such as *Gallotia galloti*, *Lacerta graeca* (*Hellenolacerta graeca*), *L. dugesii* (*Teira dugesii*), *L. pater* (*Timon pater*), *L. lepida* (*Timon lepidus*), *L. trilineata*, *L. schreiberi*, *L. viridis* (Figure 5). The adrenal gland appears rather compact in all these species except for *L. pater*, where it is elongated; moreover, the gland is surrounded by a capsule of connective tissue, that is thin in all these species except for *L. lepida*, where it is thick. The steroidogenic tissue has prismatic cells with a roundish nucleus; the tissue is organized to form sinuous anastomosing cords of two cell rows, separated by small blood vessels. In *L. dugesii*, the nucleus is placed at basal pole, outlining the shape of the steroidogenic cords. The chromaffin tissue varies in position and forms a compact ribbon made up by many rows of cells. This ribbon is placed on the dorsal margin of the gland in *L. lepida* (*T. lepidus*), *L. trilineata*, *L. schreiberi*, *L. viridis*; it sends digitations between the steroidogenic cords, a characteristic indicating a greater degree of contact between the two types of tissue. In *G. galloti*, *L. graeca* (*H. graeca*) and in *L. pater* (*T. pater*), the chromaffin tissue is placed at the cephalic pole of the gland, whereas it is placed at the two poles of the gland in *L. dugesii* (*T. dugesii*). In these three species, the dorsal ribbon is devoid of digitations between the steroidogenic tissue, a structural organization indicating a high degree of separation between the two types of tissue. The chromaffin tissue also forms islets scattered in the steroidogenic parenchyma, that are lacking in *L. graeca* (*H. graeca*), rare and of small size in *L. dugesii* (*T. dugesii*), *L. pater* (*T. pater*), *L. lepida* (*T. lepidus*), numerous and of small size in *L. trilineata*, numerous and of medium size in *L. schreiberi*, few and of medium size in *L. viridis* (Figure 5). The islets only contain E cells.

**Scheme 1:**
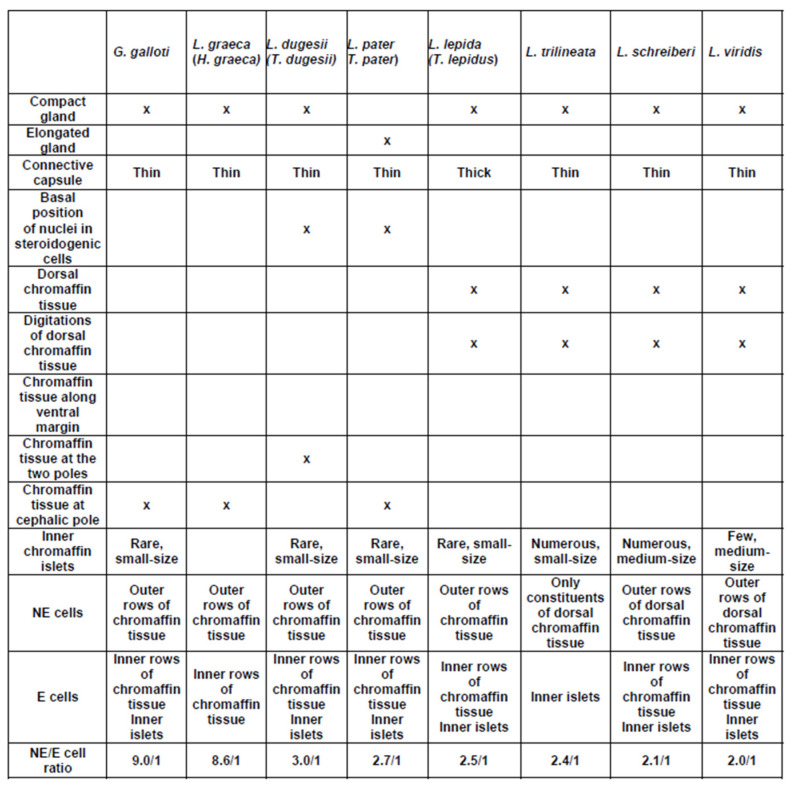
Schematic summary of the main characteristics of the adrenal gland of some Lacertids and their NE/E cell ratio. x: presence of the characteristic described on the left.

As regards the composition of the chromaffin ribbon, the NE cells are the only components in *L. trilineata*, whereas in the other species, they are present in the outer rows of the chromaffin ribbon. The E cells are present instead in the inner rows of the chromaffin ribbon and in its digitations between the steroidogenic cords, in addition to the chromaffin islets. The different distribution of the NE and the E cells affects the NE/E cell ratio, whose values are given in Scheme 1, together with a summary of the main characteristics of the gland in the examined species.

**Figure 6:**
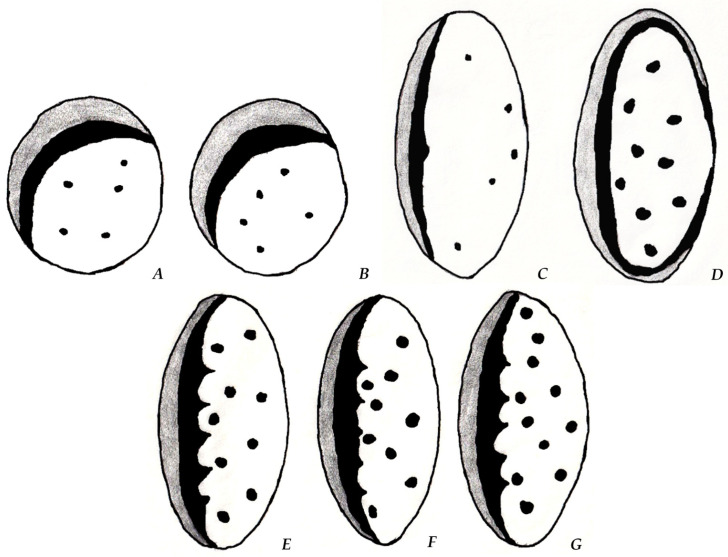
Schematic drawing showing the different organization of the NE cells (grey), the E cells (black) and the steroidogenic tissue (white) in the adrenal gland of (**A**) *M. suborbitalis*; (**B**) *M. cuneirostris*; (**C**) *A. erythrurus*; (**D**) *H. lugubris*; (**E**) *P. husabensis*); (**F**) *P. namaquensis*; (**G**) *P. undata*.

Another example of the variability of the adrenal gland can be provided by the examination of a group of african lacertids: *Meroles suborbitalis*, *Meroles cuneirostris*, *Acanthodactylus erythrurus*, *Heliobolus lugubris*, *Pedioplanis husabensis*, *Pedioplanis namaquensis*, *Pedioplanis undata* [72] (Figure 6). The adrenal gland appears elongated except for *M. suborbitalis* and *M. cuneirostris*, where it is compact; moreover, the gland is surrounded by a thin connective capsule. The steroidogenic tissue is organized to form sinuous anastomosing cords of two cell rows; the nuclei are basally displaced in *P. husabensis*, *P. namaquensis* and *P. undata.* The chromaffin tissue is more concentrated at the cephalic pole but also extends on the dorsal margin of the gland in *M. suborbitalis* and *M. cuneirostris*. The organization of the chromaffin tissue in other species is different, because in *H. lugubris* it is present on the dorsal margin, where it forms a thick ribbon that also extends to the ventral margin, where, however, it is thinner. In *A. erythrurus*, the chromaffin tissue forms a thin ribbon on the whole dorsal margin of the gland, whereas in the three specie of *Pedioplanis* the chromaffin tissue forms a rather thick dorsal ribbon sending digitations between the steroidogenic cords. The chromaffin islets, containing E cells, are rare and small in all the species except for *H. lugubris* and the three *Pedioplanis*, where they are numerous and of large size. About the composition of the chromaffin ribbon, the NE cells are present in the outer rows of the chromaffin ribbon, whereas the E cells are present in the inner rows of the chromaffin ribbon and in its digitations between the steroidogenic cords, in addition to the chromaffin islets (Figure 6).

**Scheme 2:**
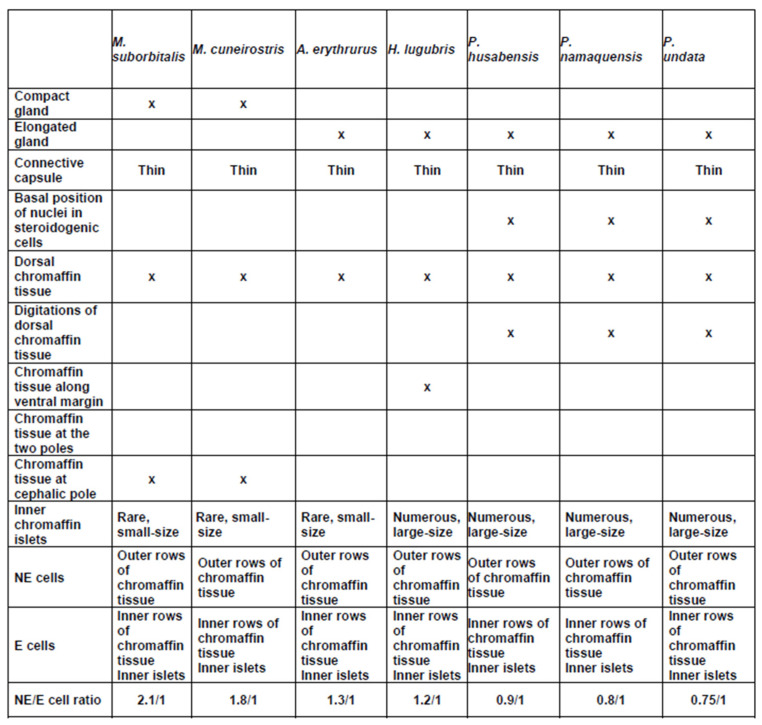
Schematic summary of the main characteristics of the adrenal gland of some Lacertids and their NE/E cell ratio. x: presence of the characteristic described on the left.

The different distribution of the NE and the E cells affects the NE/E cell ratio, whose values are given in Scheme 2, together with a summary of the main characteristics of the gland in the examined species.

**Figure 7:**
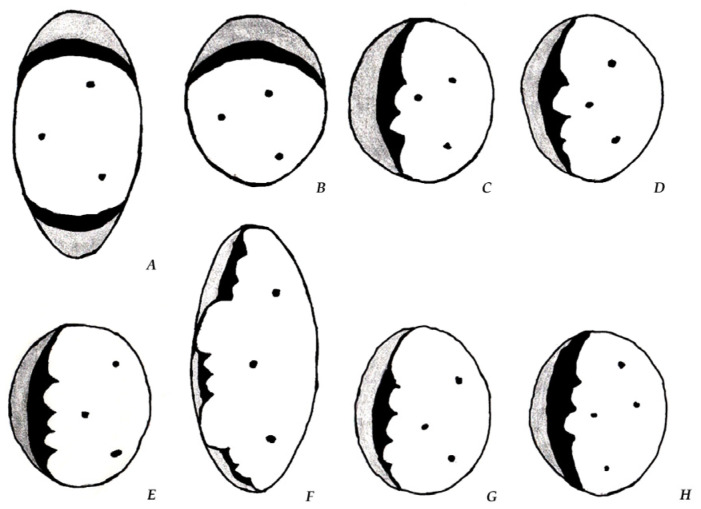
Schematic drawing showing the different organization of the NE cells (grey), the E cells (black) and the steroidogenic tissue (white) in the adrenal gland of (**A**) *P. taurica* (*P. tauricus*), (**B**) *P. hispanica* (*P. hispanicus*), (**C**) *P. wagleriana* (*P. waglerianus*), (**D**) *P. muralis*, (**E**) *P. s. sicula* (*P. s. siculus*), (**F**) *P. peloponnesiaca* (*P. peloponnesiacus*), (**G**) *P. s. klemmeri*, (**H**) *P. melisellensis*.

The existence of interspecific variations within the same genus is also confirmed by the study of a group of lizards of the genus *Podarcis*: *P. taurica* (*P. tauricus*), *P. hispanica* (*P. hispanicus*), *P. wagleriana* (*P. waglerianus*), *P. muralis*, *P. s. sicula* (*P. s. siculus*), *P. peloponnesiaca* (*P. peloponnesiacus*), *P. s. klemmeri*, *P. melisellensis* [73]. As can be seen from Figure 7, the adrenal gland is elongated in *P. taurica* (*P. tauricus*) and very elongated in *P. peloponnesiaca* (*P. peloponnesiacus*), whereas the other species have a compact gland. This gland is surrounded by a thin connective capsule that only in *P. s. klemmeri* has a greater thickness. In the steroidogenic cells, the nuclei have a basal position in *P. taurica* (*P. tauricus*) and *P. hispanica* (*P. hispanicus*). The chromaffin tissue is located at the two poles of the gland in *P*. *taurica* (*P. tauricus*) and at the head pole in *P. hispanica* (*P. hispanicus*). In the other species, this tissue forms on the dorsal pole of the gland a ribbon that in *P. peloponnesiaca* (*P. peloponnesiacus*) is discontinuous, perhaps because of extreme length of the gland; the dorsal ribbon sends digitations between the steroidogenic cords. About the composition of the chromaffin ribbon, the NE cells are present in the outer rows of the chromaffin ribbon, whereas the E cells are present in the inner rows of the chromaffin ribbon and in its digitations between the steroidogenic cords, in addition to the chromaffin islets present in all these species (Figure 7).

**Scheme 3:**
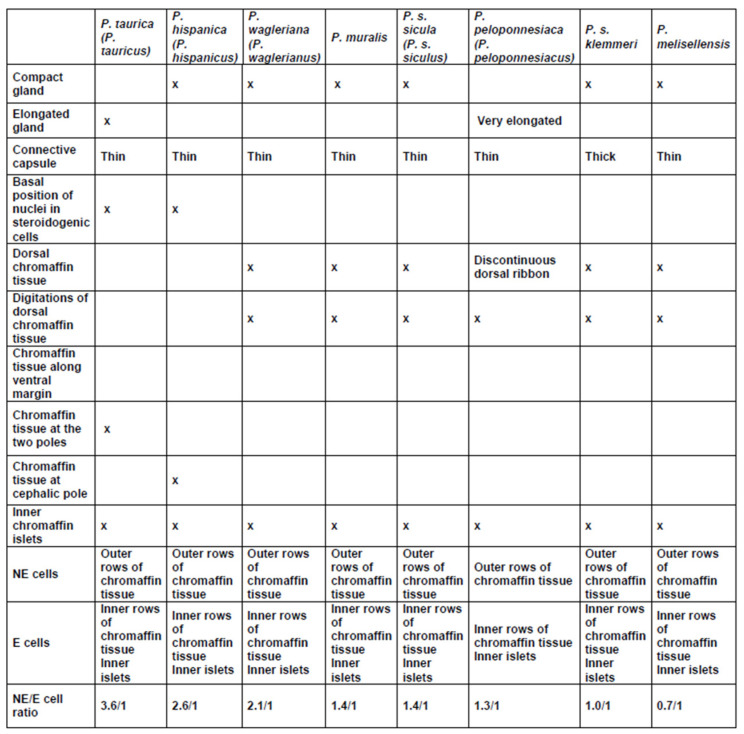
Schematic summary of the main characteristics of the adrenal gland of some Lacertids and their NE/E cell ratio. x: presence of the characteristic described on the left.

Also in this case, the different distribution of the NE and the E cells affects the NE/E cell ratio, whose values are given in Scheme 3, together with a summary of the main characteristics of the gland in the examined species.

**Scheme 4:**
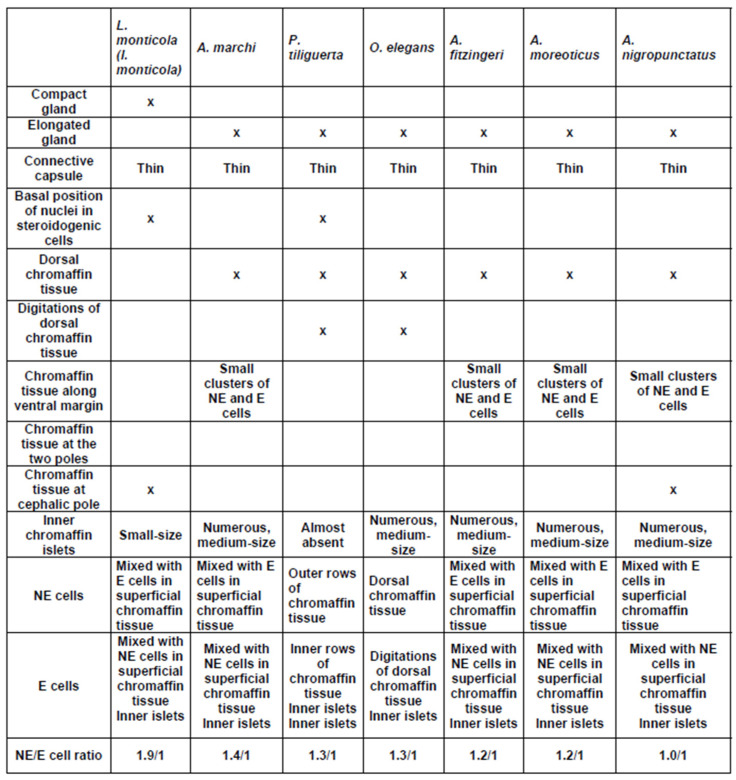
Schematic summary of the main characteristics of the adrenal gland of some Lacertids and their NE/E cell ratio. x: presence of the characteristic described on the left.

**Scheme 5:**
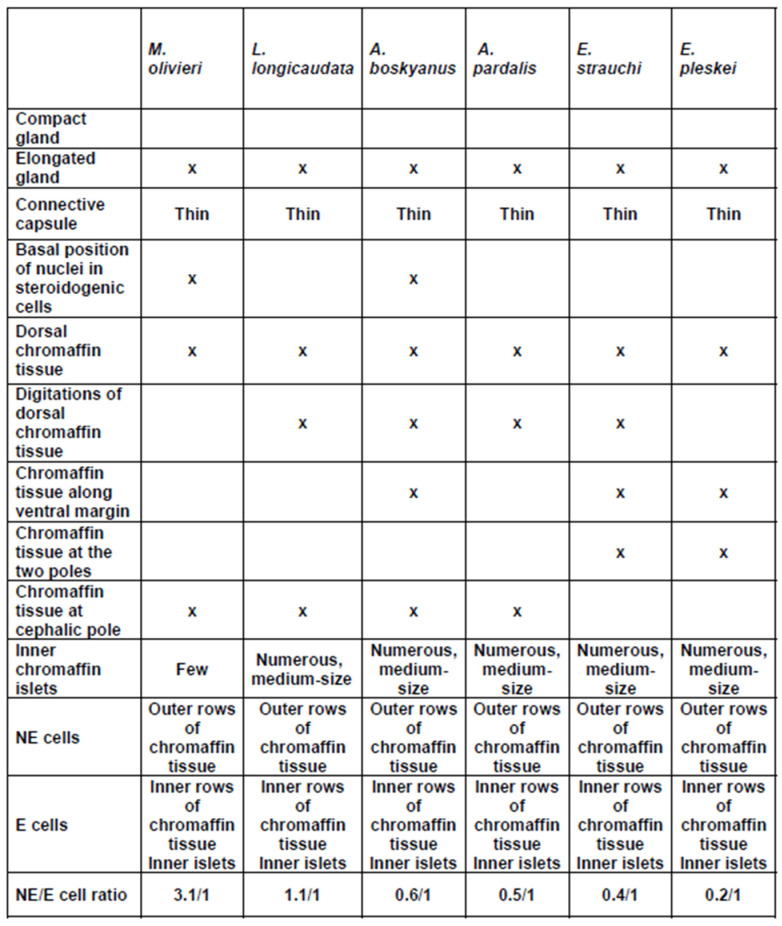
Schematic summary of the main characteristics of the adrenal gland of some Lacertids and their NE/E cell ratio. x: presence of the characteristic described on the left.

Many other species have been examined, including *Lacerta monticola* (*Iberolacerta monticola*), *Algyroides marchi*, *Podarcis tiliguerta*, *Ophisops elegans*, *A. fitzingeri*, *A. moreoticus*, *A. nigropunctatus* [2,74]; *Mesalina olivieri*, *Latastia longicaudata*, *Acanthodactylus boskyanus*, *A. pardalis*, *Eremias strauchi*, *E. pleskei* [2,75], whose main characteristics are reported in Scheme 4 and Scheme 5, respectively.

### 6.2. Scincidae

The family Scincidae is the largest lizard family, with over 100 genera and 1300 species; skinks occur on all continents except Antarctica as well as on many oceanic islands [76]. The adrenal morphology has been studied in many species including *Chalcides chalcides vittatus*, *Eumeces inexpectatus* (*Plestiodon inexpectatus*), *Tiliqua gigas*, *Eumeces obsoletus* (*Plestiodon obsoletus*), *Eumeces schneideri*, *Scincus scincus* [77]. Also, in this case there are differences in the morphology of the adrenal gland, that in all the species is elongated and surrounded by a thin connective tissue. The steroidogenic tissue is organized in anastomosing cords of two cell rows, with basal nuclei. The chromaffin tissue is placed on the dorsal margin of the gland, forming a ribbon having digitations that deepens between the steroidogenic cords; moreover, between the cords are present small islets of E cells. Small groups of chromaffin cells, having NE and E cells, are present also on the ventral surface of the gland. The organization of the adrenal gland is different in *E. schneideri* and *T. gigas*. In *E. schneideri*, the chromaffin tissue forms a continuous envelope covering all the gland, whereas, in *T. gigas* it is mainly present at the two poles of the gland. In all the species, the NE cells occupy the outer layers of the dorsal ribbon or of the envelope, while the E cells occupy the inner layers and their digitations. The chromaffin islets only contain E cells; in *T. gigas* they are almost absent whereas in the other species they are numerous and of medium size. The values of the NE/E cell ratio range from 5.8/1 of *Chalcides chalcides vittatus* to 0.37/1 of *S. scincus* [77].

### 6.3. Superfamily Cordyliformes

The superfamily Cordyliformes is a clade of scincomorph lizards having two families: Cordylidae, living in the sub-Saharan Africa, and Gerrhosauridae, also living in Madagascar [78]; different genera belonging to this superfamily were examined [79]. For example, in the genus *Gerrhosaurus*, belonging to the family Gerrhosauridae, the steroidogenic parenchyma is surrounded by an almost continuous chromaffin envelope, made up by a single layer. The envelope has NE cells, whereas the E cells are present in the rare digitations of the envelope and are few. The interrenal islets are absent; the values of the NE/E cell ratio are high, around 6.0/1. The chromaffin tissue of the genus *Platysaurus* (Cordylidae) forms a discontinuous envelope, of two or more layers, around the gland; moreover, some inner islets are present between the steroidogenic cords. The NE and the E cells are present in the envelope and in the islets; the value of NE/E cell ratio is around 3.5/1. In the genus *Pseudocordylus* (Cordylidae) the adrenal gland has a dorsal chromaffin ribbon; only few clusters of chromaffin cells are present on the ventral surface of the gland. The chromaffin ribbon is mainly made up by NE cells, and some E cells are present in the digitations of the ribbon. The chromaffin islets, containing E cells, are rare; the value of the NE/E cell ratio is around 3.3/1. In the genus *Cordylus* (Cordylidae), the chromaffin tissue is present on the dorsal region of the gland, although with some differences between the various species, concerning the thickness of the dorsal ribbon, its extension on the steroidogenic parenchyma, the presence of interrenal islets and the distribution of the NE and the E cells. The value of NE/E cell ratio ranges from 3.5/1 to 1.2/1.

## 7. The NE/E Cell Ratio and the Distribution of the Chromaffin Cells in Squamata

From studies performed on many different species of reptiles, belonging to different infraorders, families, and genera, the adrenal gland has an uneven morphology with this variability found in different species belonging to all infraorders of reptiles [2]. The adrenal gland can be compact or elongated; may be surrounded by a thin or thick connective capsule; the nuclei of steroidogenic cells may have a basal position, to precisely delineate the shape of the steroidogenic cords, or not. The relationships between the steroidogenic and the chromaffin tissues can be very different. The two tissues can come in contact as two separate regions, as when the chromaffin tissue is present at the two poles, or at head pole, of the gland, or when it forms an envelope that surrounds the steroidogenic parenchyma in part, or completely. In contrast, the two tissues can establish a greater degree of interaction as when a compact dorsal ribbon sends digitations between the steroidogenic cords, and the chromaffin islets are interspersed in the steroidogenic parenchyma. Finally, the distribution of the NE and the E cells is variable; in general, the NE cells occupy the outer layers of the superficial chromaffin tissue, whereas the E cells occupy the inner layers and its digitations, when they are present, and constitute inner islets. However, in some cases the NE and the E cells are mixed, with no preferential distribution.

However, there are also significant changes in the value of the NE/E cell ratio. The values of this ratio are very variable and range from 9.0/1 of *G. galloti* to 0.7/1 of *P. melisellensis*; these differences concern not only different families, but also species belonging to the same family or subfamily or the same genus [2]. The variations of the NE/E cell ratio correspond to the degree of contact between the two types of tissue; indeed, when the steroidogenic and the chromaffin tissues are separated, as in *G. galloti* and *L. graeca* (*H. graeca*), the values of this ratio are high, 9.0/1 and 8.6/1, respectively. When instead there is a high degree of contact between the steroidogenic and the chromaffin tissues, the values of the ratio are generally low, around 1/1. It is believed that the reason for this correspondence is the activation of the enzyme PNMT by adrenal glucocorticoids, demonstrated also in reptiles [24,79,80]. When the degree of contact between the two types of tissue is high, the chromaffin cells are exposed to high levels of corticosteroids, sufficient to activate the PNMT enzyme, whereas when the two tissues are separated, the exposure of chromaffin cells to corticosteroids is very low. As a matter of fact, when the values of NE/E cell ratio are low, the degree of contact between the steroidogenic and the chromaffin tissues is high, and the E cells are present in the regions of the chromaffin tissue closer to the steroidogenic parenchyma, as the inner layers of the superficial chromaffin ribbon, its digitations, and the inner islets. Instead, when the values of NE/E cell ratio are high, the degree of contact between the steroidogenic and the chromaffin tissues is low, and the chromaffin tissue is concentrated in large masses, so that only few cells are in contact with the steroidogenic parenchyma.

The comparison of the distribution of the chromaffin cells in the adrenal gland of Squamata with the evolution of the adrenal gland in other vertebrates, suggested the existence of a relationship between the content of NE and E and the phylogenesis. From this point of view, the species with a higher NE content were considered of more primitive ancestry, while species with a higher E content were considered recently evolved species [2]. In the species studied, such relationship was confirmed by paleontological, karyological and biochemical data [53,54,55,56]. For example, taxonomic and phylogenetic analyses of the genus *Lacerta* led to a former division of this genus into the following groups: *Lacerta* part I, *Lacerta* part II, *Podarcis* and *Gallotia* [53,54,55,56]. *Lacerta galloti* (quoted in this review as *Gallotia galloti*) was ascribed to the genus *Gallotia*, that according to biochemical data, was considered an ancient group that separated very early from the other ones [56,81,82]. In agreement with this statement, the adrenal gland of *G. galloti* is characterized by a high degree of separation between the steroidogenic and the chromaffin tissues, and a high value of the NE/E cell ratio (9.0/1), as can be expected for a species of more ancient origin. Also *L. graeca* (*H. graeca*) has a high value of NE/E cell ratio (8.6/1) and its adrenal gland is characterized by a high degree of separation between the steroidogenic and the chromaffin tissues. Arnold, indeed [53], included this species in the group *Lacerta* part II, formed by ancient lizards. Later, genetic analyses revealed that the family Lacertidae has two subfamilies, Gallotiinae and Lacertinae; the latter includes two monophyletic tribes, the Eremiadini of Africa and arid southwestern and central Asia, and the Lacertini of Europe, northwestern Africa, and southwestern and eastern Asia [81]. Currently, eight species are included in the genus Lacerta, whose systematics still seem in question [83].

Another example can be provided by the species belonging to the genus *Podarcis*, considered by Arnold [53] of recent origin [2]. The adrenal gland of most of the species belonging to this genus has a rather uniform organization, a similar distribution of the steroidogenic and the chromaffin cells, and low values of the NE/E cell ratio, except for *P. hispanica* (*P. hispanicus*) and *P. taurica* (*P. tauricus*), in which the values of the NE/E cell ratio are high (2.6/1 and 3.6/1, respectively), and indicate a high degree of separation between the two adrenal tissues. *P. hispanica* (*P. hispanicus*) was considered by Arnold [53] one of the more primitive members of the group *Podarcis*, which accords with the type of morphology of the gland and the value of NE/E cell ratio; the same should be true for *P. taurica* (*P. tauricus*), which shows the highest value of NE/E cell ratio (3.6/1) found in the *Podarcis* studied [2]. The genus *Podarcis*, whose taxonomy is complex and unstable, is characterized by a uniform morphology, so that few characters are useful for phylogenetic analyses. According to Harris e Arnold [84], the genus *Podarcis* includes four main groups characterized by substantial geographic coherence: Western Island group, southwestern group, that includes *P. hispanica* (*P. hispanicus*), Italian group, and Balkan Peninsula group, that includes *P. taurica* (*P. tauricus*). Studies performed using partial mtDNA sequences for cytochrome b (cyt b) and 16S rRNA (16S) have supported the monophyly of *Podarcis* of Balkan Peninsula group and suggest the presence of three phylogenetic clades: the clade A (*P. taurica*, *P. gaigeae*, *P. milensis*, and *P. melisellensis*); the clade B (*P. erhardii* and *P. peloponnesiaca*), and the clade C (*P. muralis* and *P. sicula*) [85]. However, due to the existence of substantial intra-specific variability, the taxonomy of *Podarcis* is continuously subject to revision [84,86], as well as the taxonomic status of *P. taurica* (*P. tauricus*) [87]. Therefore, as mentioned above, the correspondence between the value of the NE/E cell ratio and the phylogenetic history should be verified.

## 8. Conclusions

The adrenal gland is a very complex gland that, during the evolution, underwent many changes in its position, organization, and relationships between its main tissues, the steroidogenic and the chromaffin tissues. In Reptiles, except for Chelonia, the gland is near the gonads and genital ducts. The morphology of this gland, the degree of contact between the two tissues, and the values of the NE/E cell ratio are different not only between different families, but also in the same family, or subfamily, or genus, and it is likely that these differences could have a phylogenetic meaning.

However, the study of the adrenal gland of the reptiles is also important from another point of view, that related to the risk of extinction that has been documented also for these organisms, besides that already known for the other tetrapods. Indeed, around 60% of the world’s turtles, almost 50% of all crocodilians and nearly 20% of lizard and snakes are at risk of extinction, with an average global decline for reptile population of around 55% between 1970 and 2012 [88]. The factors that threaten the survival of reptiles are the same that threaten the survival of other tetrapods: deforestation, agriculture, urbanization, and the endocrine disruptors that accumulate progressively in the environment. Reptiles, indeed, have a wide geographic distribution, are long-lived species, tend to stay in the same environment and are very sensitive to the environmental pollutants, that these organisms may accumulate and biomagnify. For all these reasons, reptiles are considered excellent bioindicators of environmental contamination [89,90,91]. The reptilian adrenal gland has proven to be a target of the effects of endocrine disruptors, that influence both the morphology and physiology of this gland. Indeed, after the exposure to many endocrine disruptors, alterations of both the chromaffin and the steroidogenic tissues, of the value of the NE/E cell ratio and of the plasma hormone levels produced by this gland have been observed. Similar changes have been found also in other vertebrate species (see [7] for review). Considering the role played by the adrenal gland in the physiology of living organisms, in this case of reptiles, it is easy to guess how harmful the repercussions of the alterations of this gland on the organism are. Therefore, the understanding of the normal state of the adrenal gland of Squamata is essential in detecting adaptative changes in response to xenobiotics, endocrine disruptors, and environmental insults of Anthropocene-Pyrocene period.
